# Prospective Evaluation of Pharmacogenomics and Metabolite Measurements upon Azathioprine Therapy in Inflammatory Bowel Disease: An Observational Study

**DOI:** 10.1097/MD.0000000000003326

**Published:** 2016-04-18

**Authors:** Zhang Fangbin, Gao Xiang, Ding Liang, Liu Hui, Wang Xueding, Chen Baili, Bi Huichang, Xiao Yinglian, Cheng Peng, Zhao Lizi, Chu Yanjun, Xu Feng, Chen Minhu, Huang Min, Hu Pinjin

**Affiliations:** From the Department of Gastroenterology, the First Affiliated Hospital of Zhengzhou University, Zhengzhou (ZF, CP, CY, XF); Department of Gastroenterology, the First Affiliated Hospital, Sun Yat-sen University (ZF, GX, CB, XY, CM, HP); Department of Gastroenterology, the Sixth Affiliated Hospital of Sun Yat-sen University (GX, HP); Institute of Clinical Pharmacology, School of Pharmaceutical Sciences, Sun Yat-sen University (DL, LH, WX, BH, ZL, HM); and Department of Pharmacology, the First Affiliated Hospital of Jinan University (LH), Guangzhou, China).

## Abstract

Up to approximately 40% to 50% of patients discontinue thiopurine therapy during the course of inflammatory bowel disease (IBD). We investigated the role of the metabolite thiopurine in IBD treatment. This was a prospective study.

IBD patients receiving azathioprine (AZA) were prospectively included. Thiopurine methyltransferase (TPMT) genotypes were examined before therapy, and thiopurine metabolite levels were examined at weeks 2, 4, 8, 12, 24, and 48. In total, 132 patients were included. The frequency of leucopenia increased at 6-thioguanine nucleotide (6-TGN) levels ≥420 pmol/8 × 10^8^ RBC (odds ratio [OR] = 7.9; 95% confidence interval (95%CI): 3.5–18.0; *P* < 0.001) and increased more during the initial 12 weeks of thiopurine therapy (OR = 16.0; 95%CI: 5.7–44.9; *P* < 0.001). The patients with 6-TGN levels ≥420 pmol/8 × 10^8^ RBC at weeks 4, 8, and 12 had an increased likelihood of leucopenia. Clinical response increased at 6-TGN levels ≥225 pmol/8 × 10^8^ RBC (OR = 13.5; 95% CI: 3.7–48.9; *P* < 0.001) in Crohn disease (CD) patients. The CD patients with 6-TGN levels ≥225 pmol/8 × 10^8^ RBC at weeks 8, 12, and 24 had an increased likelihood of successful clinical response. TPMT∗3C had a specificity of 100%, but a sensitivity of 8% for predicting leucopenia.

A 6-TGN level between 225 and 420 pmol/8 × 10^8^ RBC could be a therapeutic window in patients receiving AZA therapy, and it could likely predict leucopenia in the initial 12 weeks of AZA therapy and a reasonable chance of successful clinical response in CD patients. The value of TPMT genotyping before thiopurine therapy is limited in Chinese patients with IBD, considering the low sensitivity of predicting leucopenia.

## INTRODUCTION

The thiopurine drugs, azathioprine (AZA) and mercaptopurine, have been widely used in the treatment of inflammatory bowel disease (IBD). They can induce and maintain remission with a response rate of 55% to 70%. However, up to approximately 40% to 50% of IBD patients discontinue thiopurine therapy during the course of the disease.^1,2^ The occurrence of intolerable adverse events (AEs) is the major reason for drawback in the thiopurine therapy, and the AEs are reported in 10% to 28% of IBD patients.^2–6^ Other reported reasons for discontinuation of thiopurines are refractoriness (16%), request by the patient (7%), and pregnancy (1%).^6^

After oral absorption, AZA is converted to 6-mercaptopurine via a nonenzymatic reaction, and 6-mercaptopurine is subsequently metabolized to active metabolites, such as 6-thioguanine nucleotides (6-TGNs) and 6-methylmercaptopurine ribonucleotides (6-MMPRs), which have immune modifier activity, and inactive metabolite: 6-methylmercaptopurine (6-MMP). Three enzymes competitively metabolize 6-mercaptopurine: xanthine oxidase, hypoxanthine phosphoribosyl transferase, and thiopurine methyltransferase (TPMT). The enzyme TPMT plays an important role in determination the amount of cytotoxic 6-TGN. Low baseline levels of TPMT activity led to a shift in metabolism of 6-mercaptopurine away from production of the inactive metabolite 6-MMP and toward production of the “active” metabolite 6-TGN.^7^

The genetic variation of TPMT and its influence on thiopurine metabolism have been well investigated. The literatures showed that individuals with TPMT deficiency who receive standard doses of thiopurines are at significant risk of toxicity, primarily as a result of unchecked production of 6-TGN.^8–11^ However, not all AEs or metabolite patterns can be explained by TPMT deficiency. A study from Madrid showed that the authors could not confirm whether the choice of AZA or a mercaptopurine dose based on TPMT activity prevented leucopenia in patients with IBD.^12^ Reports have suggested that variation in TPMT activity accounted for no >10% of overall thiopurine toxicity and approximately one-third of cases of leucopenia.^13^

Considerable interest has focused on the metabolism of thiopurines as a means of identifying ways of individualizing therapy to minimize AEs and maximize clinical responses. Previous retrospective meta-analysis studies and cross-sectional studies have confirmed a strong association between 6-TGN concentrations and induction of remission.^14^ These studies showed that a 6-TGN level consistently above 230 to 260 pmol/8 × 10^8^ RBC was associated with a favorable response.^14^ However, not all related studies have demonstrated an association between higher 6-TGN concentrations and improvement in disease activity.^15–17^ Meanwhile, not all leucopenia can be explained by a high 6-TGN concentration in the IBD patients.^15,18–20^

Given this background, the aims of this study were to evaluate the role of TPMT polymorphisms before treatment, metabolic products of AZA, and disease activity and toxicity in patients with IBD during their first 48 weeks of thiopurine therapy.

## MATERIALS AND METHODS

### Subjects

All patients with IBD between August 2006 and August 2014 at the Gastroenterology Clinic of the First Affiliated Hospital of Zhengzhou University and Sun Yat-sen University were included in our study. The diagnoses of IBD were according to the criteria of Lennard-Jones,^21^ which are based on clinical features and endoscopic, radiological, and histopathological findings.

The inclusion criteria and exclusion criteria were defined as described previously.^5^

### Study Design

Drug doses were initiated at 50 mg daily for AZA (Imurel®, GlaxoSmithKline, Sweden) and 25 mg daily for mercaptopurine (Puri-Nethol®, GlaxoSmithKline) in the first week, and the target doses of nearly 2.0 mg/kg daily for AZA and 1.0 mg/kg daily for mercaptopurine were reached by week 2 without alteration in the following weeks.

Patient data collection and study visits were performed as described previously.^5^ TPMT genotype was measured before initiation of therapy. Blood was drawn at weeks 1, 2, 3, 4, 8, 12, 24, and 48 for future analysis of 6-TGN and 6-MMPRs.

The primary endpoint of the study was defined as the patient undergoing AZA treatment for 48 weeks or requiring discontinuation or change of therapy because of AEs or refractoriness.

### Classification of Responses

To evaluate the relationship between response to steroid sparing and metabolite levels, the patients receiving AZA plus infliximab, receiving AZA for postoperative prophylaxis, and receiving AZA for endoscopic relapse after surgical resection were excluded from the analysis. A response to steroid sparing was defined as resolution of symptoms upon complete withdrawal of all corticosteroids for at least 6 months (CDAI <150,^22^ or Southland Index ≤2).^23^ Treatment failure was ascribed to cases in which the above-stated therapeutic goals were not achieved and there was need for initiation of other immunosuppressive medications or surgery.

### TPMT Genotyping

Allele-specific polymerase chain reaction (PCR) and PCR-restriction fragment length polymorphisms were used to determine the frequency of TPMT mutant alleles (ie, TPMT∗2, TPMT∗3A, TPMT∗3B, and TPMT∗3C) in patients with IBD, as previously described.^5^

### Erythrocyte Mercaptopurine Metabolite Assay

Erythrocyte lysis buffer was prepared as previously described.^24^ The supernatant was kept at 80°C until analysis.

The 6-TGN concentration in the erythrocyte lysates was determined using a previously described high-performance liquid chromatography (HPLC) method.^25^ The concentration of 6-TGN was normalized to pmol/8 × 10^8^ RBC. 6-MMPRs (pmol/8 × 10^8^ RBC) levels were determined by a reverse-phase HPLC assay developed by Lennard and Singleton.^26^

### Statistics

Statistical analysis was performed using SPSS (Statistical Package for the Social Sciences) software (SPSS Inc, Chicago, IL), version 16.0. Normality was tested for all quantitative variables. Quantitative variables are described as the median and the interquartile range (IQR; q1–q3) throughout. Parametric Student *t* tests or nonparametric Mann-Whitney *U* tests, when appropriate, were used to compare quantitative variables between the 2 groups. The differences between multiple independent groups were evaluated by the Kruskal–Wallis test. The *χ*^2^ test was used to compare categorical variables. To evaluate the relationship between erythrocyte AZA metabolite concentrations and the AEs, binary logistic regression was used, and the results were expressed as the odds ratio (OR) with a 95% confidence interval (95% CI). Receiver-operating characteristic (ROC) curves were obtained to plot the sensitivity and specificity for various metabolite concentrations to predict the AEs and clinical responses. A *P* value <0.05 was considered significant.

### Ethical Considerations

The study protocol was approved by the ethics committee of the First Affiliated Hospital of Zhengzhou University and Sun Yat-sen University. Each patient provided written informed consent before entering the trial. The study was registered in the International Standard Randomized Controlled Trial Number Register (ISRCTN), with a trial number of ISRCTN58287360.

## RESULTS

### Subject Characteristics

One hundred and seventy-two patients consented to participate in this study. All patients in this study are ethnic Chinese. Forty subjects were subsequently withdrawn as a result of the administration of AZA or mercaptopurine within 3 months before treatment initiation (n = 22), poor compliance (n = 7), loss of contact (n = 3), and follow-up time <48 weeks (n = 8). The remaining 132 subjects (76 men) had a mean age of 34 years (18–72 years). The majority of subjects (77%) had Crohn disease (CD), with the remainder having ulcerative colitis (UC) (23%). At the study initiation, 47 patients were classified as steroid-dependent, 49 as being on remission maintenance, 24 as having postoperative recurrence of clinical symptoms, and 12 as being on postoperative prophylaxis. Of the 132 patients, 31 patients (23%) were treated with AZA and 5-aminosalicylates (5-ASA) for >4 weeks, and 12 patients (9%) were treated with AZA and infliximab. Table 1 shows the demographic characteristics of the 132 patients.

**TABLE 1:**
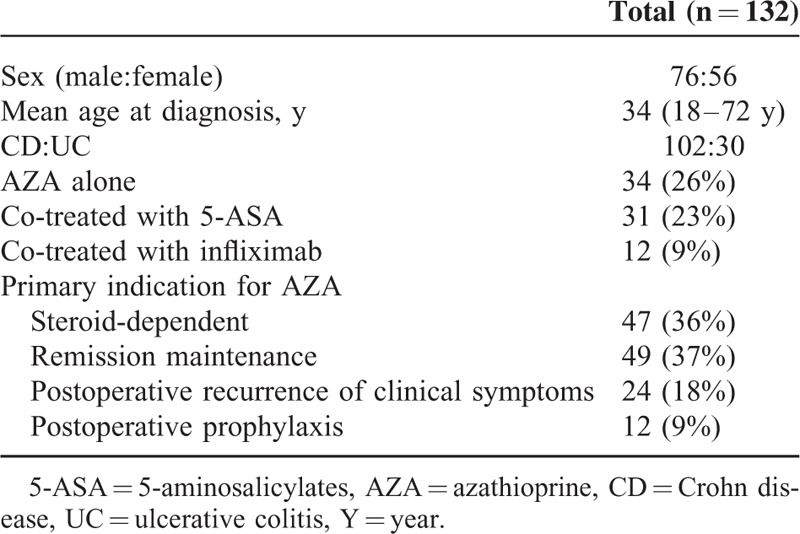
Demographic Characteristics of the Study Patients

### Adverse Effects

Twenty-eight of the 132 patients (21%) exhibited AEs during therapy. Of the 28 patients, 26 patients (20%) exhibited leucopenia. Of the 26 patients, 19 (73%) exhibited leucopenia during the initial 3 months of thiopurine therapy, 3 patients (2%) exhibited gastric intolerance, 1 patient (0.8%) exhibited flu-like symptoms, and no patient exhibited hepatotoxicity or pancreatitis. The emergence of AEs led to discontinuation of thiopurine therapy in 16 patients (57%). After discontinuation, 11 patients were reintroduced to AZA at half the original dose, 9 patients were shifted to mercaptopurine, 3 patients were shifted to MTX, and 3 patients were shifted to infliximab. Table 2 shows the details of AEs in the 28 patients.

**TABLE 2:**
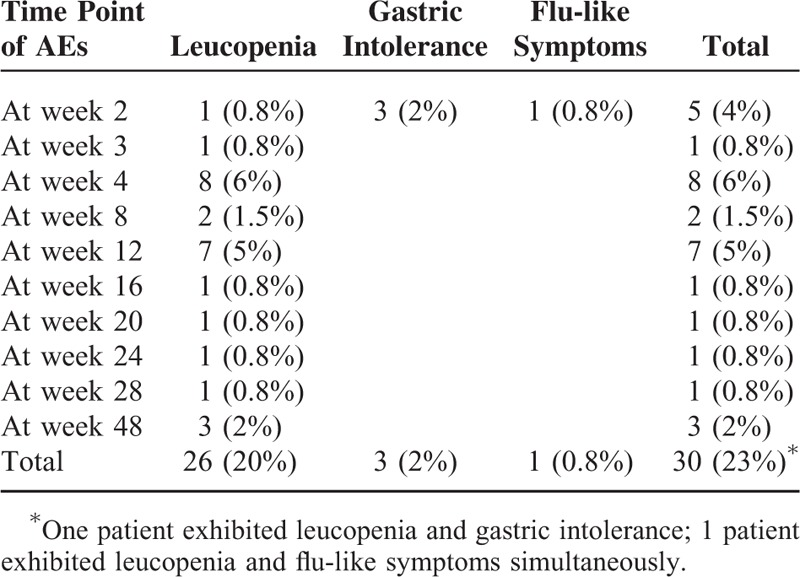
Details of Adverse Effects in 28 Patients

### Metabolite Assays and Leucopenia

**FIGURE 1:**
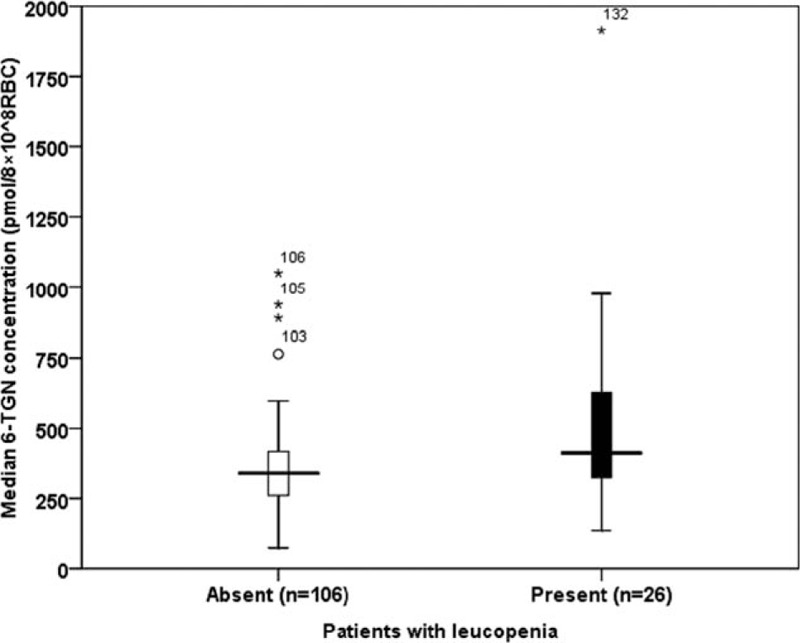
Metabolite assays and leucopenia. Median (quartile 1; quartile 3) levels of 6-TGN during the 20 week course of the study in the patients with leucopenia (n = 106) and in the patients without leucopenia (n = 26). 6-TGN = 6-thioguanine nucleotide.

Twenty-six of the 132 patients (20%) developed higher maximum 6-TGN concentrations at the point of leucopenia compared with maximum 6-TGN concentrations in the remaining 106 patients without leucopenia (412 [IQR, 317–662] vs 339 [259–418] pmol/8 × 10^8^ RBC, *P* = 0.02; Figure 1).

We undertook a logistic regression analysis to elucidate factors associated with leucopenia. The independent variables were age, sex, 5-ASA treatment, disease type (CD/UC), maximum 6-TGN (log max 6-TGN) level, and 6-MMPR (log max 6-MMPR) level; the dependent variable was leucopenia. In this regression model, only maximum 6-TGN concentration was significantly associated with leucopenia (OR = 54.4 [95% CI: 2.5–1183.0], *P* = 0.01).

The optimal cutoff for the 6-TGN levels to predict leucopenia was chosen by ROC analysis. The area under the ROC curve was 0.78 (95% CI: 0.68–0.88, *P* < .001). The calculated cutoff level was 420 pmol/8 × 10^8^ RBC with a high test specificity of 89% (395/445) and a negative prediction value of 97% (395/408), but it occurred at the expense of a sensitivity of 50% (13/26) and a positive predictive value of 21% (13/63). Patients with a 6-TGN level exceeding 420 pmol/8 × 10^8^ RBC had an increased risk of developing leucopenia (21% vs 3%, OR = 7.9 [95% CI: 3.5–18.0], *P* < 0.001). Further analysis found that the risk of suffering from leucopenia in the patients with a 6-TGN level exceeding 420 pmol/8 × 10^8^ RBC was greater during the initial 12 weeks of thiopurine therapy (27% vs 2%, OR = 16.0 [95% CI: 5.7–44.9], P < .001), and a specificity of 88% (259/294), a negative prediction value of 98% (259/265), a sensitivity of 68% (13/19), and a positive predictive value of 27% (13/48) for predicting leucopenia in the initial 12 weeks of thiopurine therapy were observed. All 7 patients with leucopenia after 12 weeks of thiopurine therapy had a 6-TGN concentration <420 pmol/8 × 10^8^ RBC at the point of leucopenia (Table 3).

**TABLE 3:**
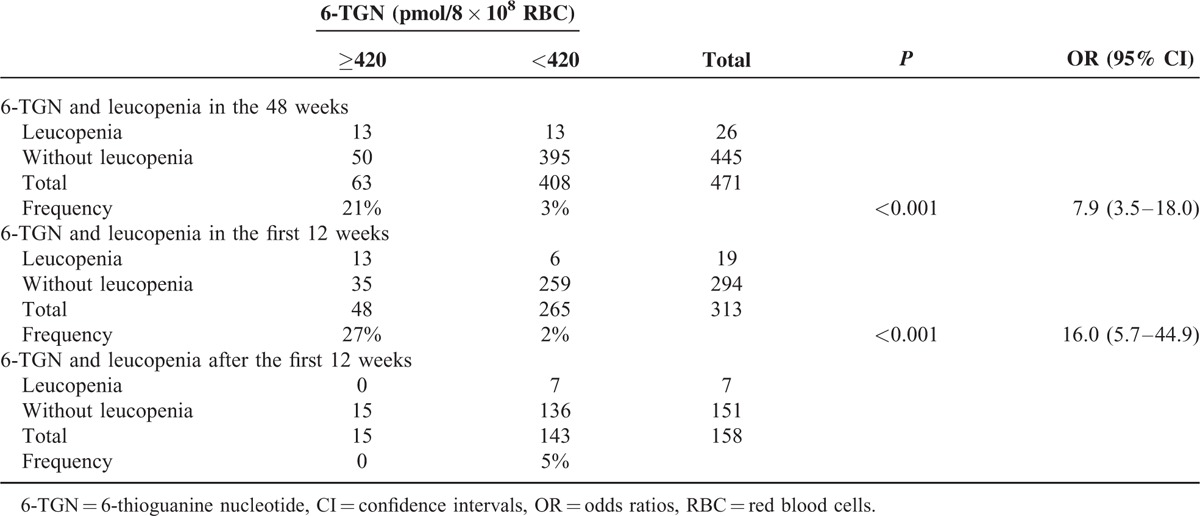
6-TGN and Leucopenia in the 471 Metabolite Measurements

### 6-TGN Concentrations at Different Weeks and Leucopenia in the Initial 12 Weeks of Thiopurine Therapy

6-TGN concentrations showed no significant differences between the patients with leucopenia at week 2 and those without leucopenia in the initial 12 weeks of thiopurine therapy (216 [IQR, 161–346] vs 204 [145–292] pmol/8 × 10^8^ RBC, *P* = 0.43). Considering that 4 patients missed the visit at weeks 4, 6-TGN concentrations at 2 weeks were combined for further analysis (the maximum 6-TGN concentration was used if a patient had 6-TGN assays at weeks 4 and 8). Concentrations of 6-TGN in the patients with leucopenia at weeks 4 to 8 were 476 (IQR, 351–766) pmol/8 × 10^8^ RBC, compared with 295 (IQR, 242–385) pmol/8 × 10^8^ RBC in those without leucopenia (*P* < 0.001). Patients with a 6-TGN level exceeding 420 pmol/8 × 10^8^ RBC at 2 weeks had an increased risk of developing leucopenia (45% vs 7%, OR = 10.3 [95% CI: 3.0–35.7], *P* < 0.001). Moreover, a specificity of 84% (62/74), a negative prediction value of 93% (62/67), a sensitivity of 67% (10/15), and a positive predictive value of 45% (10/22) for predicting leucopenia with thiopurine therapy at weeks 4 to 12 were observed based on the calculated cutoff level of 420 pmol/8 × 10^8^ RBC. The concentration of 6-TGN in the patients with leucopenia at week 12 was 436 (IQR, 226–623) pmol/8 × 10^8^ RBC, compared with 248 (IQR, 203–379) pmol/8 × 10^8^ RBC in those without leucopenia in the initial 12 weeks of thiopurine therapy (*P* = 0.04). Patients with a 6-TGN level exceeding 420 pmol/8 × 10^8^ RBC at week 12 had an increased risk of developing leucopenia (25% vs 5%, OR = 6.4 [95% CI: 1.3–32.6], *P* = 0.04). Moreover, a specificity of 83% (58/70), a negative prediction value of 95% (58/61), a sensitivity of 57% (4/7), and a positive predictive value of 25% (4/16) for predicting leucopenia of thiopurine therapy at week 12 were observed based on the calculated cutoff level of 420 pmol/8 × 10^8^ RBC (Table 4).

**TABLE 4:**
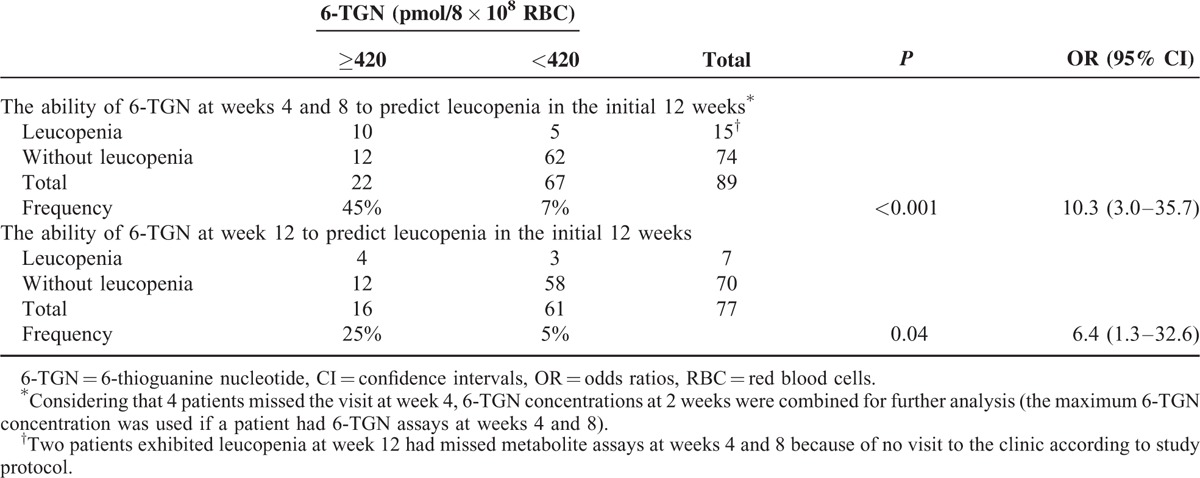
The Ability of 6-TGN at Different Weeks to Predict Leucopenia in the Initial 12 Weeks of Thiopurine Therapy

### Metabolite Assays and Other Adverse Effects

Three patients developed gastric intolerance and 1 patient developed flu-like symptoms at week 2. The 6-TGN concentrations of the 3 patients with gastric intolerance were 288 pmol/8 × 10^8^ RBC, 199 pmol/8 × 10^8^ RBC, and 193 pmol/8 × 10^8^ RBC, and the 6-MMPR concentrations were 762 pmol/8 × 10^8^ RBC, 740 pmol/8 × 10^8^ RBC, and 981 pmol/8 × 10^8^ RBC. The 6-TGN concentration of the patient with flu-like symptoms was 783 pmol/8 × 10^8^ RBC, and the 6-MMPR concentration was 2692 pmol/8 × 10^8^ RBC. One of the 3 patients with gastric intolerance and the patient with flu-like symptoms were changed to mercaptopurine with a dosage of 0.5 mg/kg daily, and both of them developed gastric intolerance and flu-like symptoms again at week 2. The 6-TGN concentrations were 234 pmol/8 × 10^8^ RBC and 83 pmol/8 × 10^8^ RBC, respectively.

### 6-TGN and Clinical Responses

To evaluate the relationship between responses to steroid sparing and 6-TGN levels, the patients receiving AZA plus infliximab (n = 12), the patients receiving AZA for postoperative prophylaxis (n = 12), and the patients whose AZA therapy was discontinued in the first 24 weeks (n = 21) because of AEs were excluded from the analysis.

Of the remaining 87 patients, 58 (8 with UC, 50 with CD) were classified as exhibiting a response to steroid sparing and 29 (9 with UC, 20 with CD) as treatment failure. In 17 patients with UC, the 6-TGN levels at weeks 8 and 24 were lower in UC patients with responses to steroid sparing than that in UC patients with treatment failure; the 6-TGN levels at week 12 were not significantly different between the 2 groups. However, the 6-TGN levels at weeks 8, 12, and 24 were higher in CD patients with responses to steroid sparing than in CD patients with treatment failure (Table 5).

**TABLE 5:**
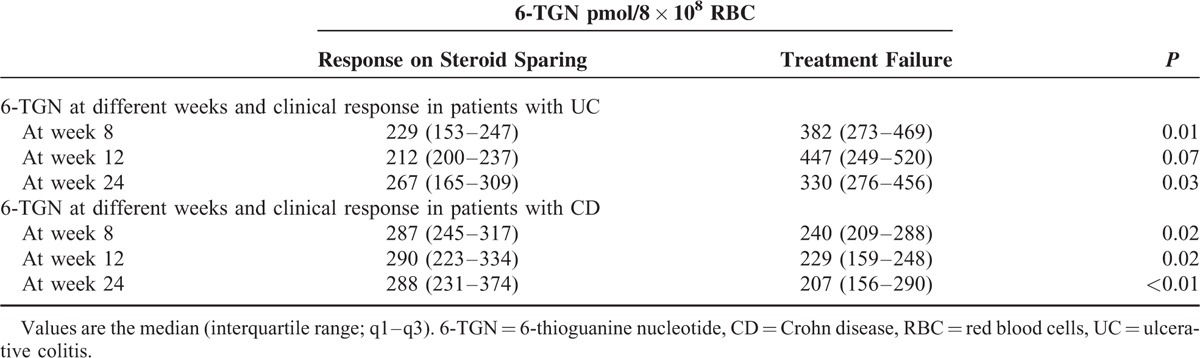
6-TGN and Clinical Responses

The optimal cutoff for the mean 6-TGN levels from week 4 to week 24 to predict a clinical response was chosen by ROC analysis. The area under the ROC curve was 0.77 (95% CI: 0.63–0.90, *P* = 0.001). The calculated cutoff level was 225 pmol/8 × 10^8^ RBC with a test sensitivity of 90% (45/50), a specificity of 60% (12/20), a positive predictive value of 85% (45/53), and a negative prediction value of 71% (12/17). The CD patients with a 6-TGN level exceeding 225 pmol/8 × 10^8^ RBC had an increased likelihood of successful clinical response (85% vs 17%, OR = 13.5 [95% CI: 3.7–48.9], *P* < 0.001).

The optimal cutoff for the 6-TGN levels at weeks 8, 12, 24 to predict a clinical response separately was chosen by ROC analysis. All the areas under the 3 ROC curves were 0.71. All the calculated cutoff levels at these weeks were: 225 pmol/8 × 10^8^ RBC with a test sensitivity of 88% (29/33), a specificity of 47% (7/15), a positive predictive value of 78% (29/37), and a negative prediction value of 64% (7/11) at week 8; a sensitivity of 76% (25/33), a specificity of 60% (9/15), a positive predictive value of 81% (25/31), and a negative prediction value of 53% (9/17) at week 12; and a sensitivity of 78% (39/50), a specificity of 60% (12/20), a positive predictive value of 83% (39/47), and a negative prediction value of 52% (12/23) at week 24. The CD patients with a 6-TGN level exceeding 225 pmol/8 × 10^8^ RBC at these weeks had an increased likelihood of successful clinical response (at week 8 OR = 6.3 [95% CI: 1.5–27.2], *P* = 0.02; at week 12, OR = 4.7 [95% CI: 1.3–17.3], *P* = 0.02; and at week 24, OR = 5.3 [95% CI: 1.7–16.3], *P* < 0.01; Table 6).

**TABLE 6:**
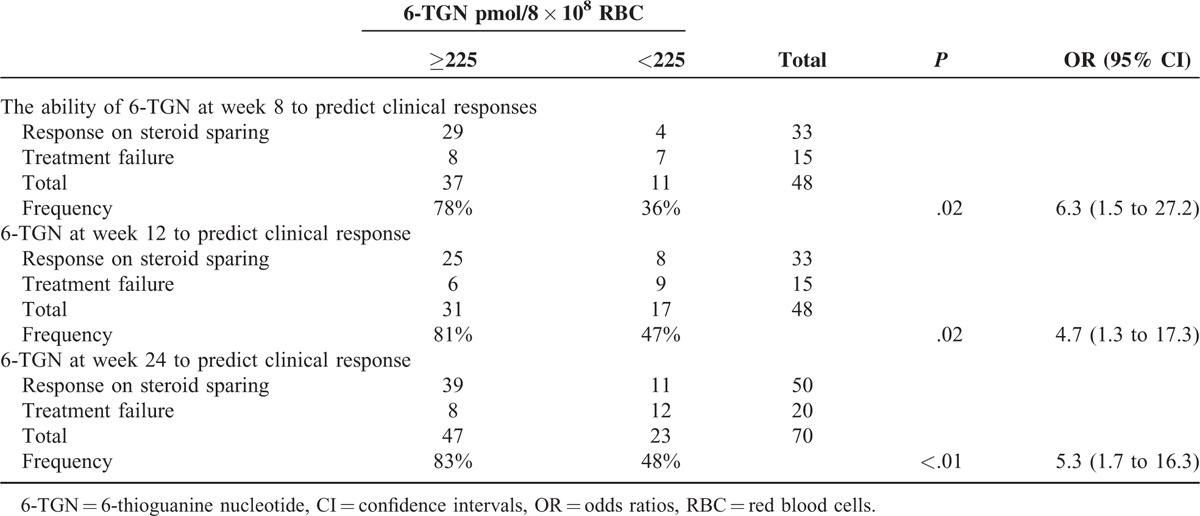
The Ability of 6-TGN at Different Weeks to Predict Clinical Responses in Crohn Disease patients

### TPMT Genotype and Leucopenia

Among the 132 patients, 2 (1.5%) were TPMT^H^/TPMT^L^ (TPMT∗3C) heterozygous. Both developed leucopenia at week 4, and the 6-TGN concentrations were 822 pmol/8 × 10^8^ RBC and 1914 pmol/8 × 10^8^ RBC. TPMT∗3C had a specificity of 100% (106/106), but at the expense of a sensitivity of 8% (2/26) for predicting leucopenia. No TPMT∗2, TPMT∗3A, or TPMT∗3B polymorphisms were detected in any of the 132 patients.

### 5-ASA and Leucopenia

Of the 132 patients, 11 of 31 patients cotreated with 5-ASA (mesalazine, 3 g daily) developed leucopenia. The frequency of leucopenia was 35% (11/31) in the patients cotreated with 5-ASA, compared with 15% (15/101) of patients not on 5-ASA (*P* = 0.01). The patients cotreated with 5-ASA had an increased likelihood of leucopenia (OR = 3.2 [95% CI: 1.3–7.9]). Of the 471 metabolite measurements, the median 6-TGN level was 315 (IQR, 236–429) pmol/8 × 10^8^ RBC in those cotreated with 5-ASA (n = 83), compared with 248 (IQR, 187–331) pmol/8 × 10^8^ RBC in those not on 5-ASA (n = 388; *P* = 0.01).

## DISCUSSION

Using a prospective study, we evaluated the use of AZA in adult patients with IBD by assessing the association between pretreatment TPMT polymorphisms, metabolic products of AZA, and disease activity and toxicity during their first 48 weeks of thiopurine therapy.

The frequency of leucopenia in this study was higher than previously reported in a Western population,^1–4,11,12,16–19^ which could be explained by the different definition of leucopenia (WBC <3.5 × 10^9^ cells/L), co-administration of 5-ASA, the rapid dose escalation practice, and the fact that the patients were followed for a longer time. The higher percentage of leucopenia could also have been a result of racial differences. The frequency of gastric intolerance was lower than the reported frequency in Western populations.^2,11,17,18^ The difference could be explained by the different definitions of gastric intolerance (the occurrence of any or a combination of the following: nausea, vomiting, and abdominal pain with normal amylase and the need to discontinue or change therapy) and racial differences.

No hepatotoxicity was reported in this study, and possible explanations include the low thiopurine dose and racial differences. Pancreatitis is thought to be an idiosyncratic reaction, and the reported frequency was 1% to 11% in Western populations.^3,4,11,17,19^ However, no pancreatitis was reported in this study or in other domestic studies,^5,27,28^ which could be explained by racial differences. The vast majority of AEs occurred during the initial 3 months of therapy, which is well in line with other observations.^29^ A substantial number (27%) of the leucopenia events developed after the initial 3 months of therapy, which is in accordance with a previous study.^5^ This observation illustrates the need for continued monitoring of blood counts throughout the duration of thiopurine therapy.

Individual variations in susceptibility to AZA have been attributed to variable intracellular concentrations of the cytotoxic 6-TGN. There is increasing evidence that 6-TGN levels above a certain threshold are associated with a higher risk of leukopenia during AZA therapy. Schütz et al^30^ reported that 2 heart recipients with a high 6-TGN level (2211 and 698 pmol/8 × 10^8^ RBCs) developed severe AZA-induced myelosuppression with a fatal outcome, and 6-TGN concentrations that were ≤450 pmol/8 × 10^8^ RBCs were not associated with myelosuppression in the remaining 18 heart recipients. Subsequently, Gupta et al^31^ and Dubinsky et al^19^ reported that leukopenia was significantly associated with high 6-TGN levels in 101 children with IBD and in 92 pediatric patients with IBD. However, Cuffari et al^18^ found that meTIMP, not 6-TGN levels, was associated with myelosuppression. However, we found that 6-TGN levels were only useful for predicting leucopenia in the initial 3 months of AZA therapy rather than after 3 months of AZA therapy. Another important finding of this study was that the delayed leukopenia in patients receiving AZA is not associated with accumulation of 6-TGN. These findings may influence the clinical application of metabolite measurements.

It has been postulated that some early idiosyncratic reactions, such as gastric intolerance and flu-like symptoms during AZA therapy, might be due to the nitro-imidazole compound found in AZA, which is released to produce 6-mercaptopurine.^32^ In accordance with the above results, all 3 patients who reacted with gastric intolerance and the one patient who reacted with flu-like symptoms in this study had low 6-TGN and 6-MMPR levels when the AEs occurred at week 2 on AZA therapy, and a re-challenge with mercaptopurine inevitably resulted in a new episode of gastric intolerance at week 2.

Several previous studies have suggested that higher levels of 6-TGN are associated with clinical responses in IBD.^1,14,19,31^ Similarly, 6-TGN levels correlated well with clinical responsiveness to therapy in our patients with CD. However, we found a lack of correlation between clinical response and 6-TGN levels in UC, which is in accordance with the study by Cuffari et al.^33^ One possible explanation might be the inconsistent or negative results of AZA for the induction of remission in chronic active UC.^34–36^ Another possible explanation might be our aggressive use of 5-ASA preparations in 15 of 17 patients with UC. Thus, our results suggested that a 6-TGN level ≥225 pmol/8 × 10^8^ RBC could be a useful therapeutic target in patients with CD. Similar results have been reported in other studies in which IBD patients in disease remission correlated well with 6-TGN levels >230 and 260 pmol/8 × 10^8^ RBC.^14^ An important finding of this study was that 6-TGN levels at weeks 8, 12, and 24 could predict a reasonable chance of successful clinical response in patients with CD.

Patient with TPMT^H^/TPMT^L^ and patient with TPMT^L^ have been demonstrated at significant risk of leucopenia when treated with standard doses of AZA or mercaptopurine.^8–10,11,37,38^ However, the sensitivity is low in using the TPMT genotype to predict leucopenia. A study from Colombel showed that three-quarters of leucopenia were not explained by *TPMT* gene mutations.^13^ Meanwhile, only 8% of patients with leucopenia had *TPMT∗3C* gene mutations in this present study, which corroborates our previous pilot study on IBD.^5^ The distribution of TPMT polymorphism is different significantly in different population. The predominant TPMT mutant allele is TPMT∗3A (3.2–5.7%) in white populations,^39–42^ followed by TPMT∗2 (0.5%) and TPMT∗3C (0.8%), and the most prevalent mutant allele is TPMT∗3C (0.5%–1.5%) in Asian populations.^5,28,43^ Thus, the value of TPMT genotype measurement before initiation of AZA was limited in predicting leucopenia because of low sensitivity.

Previous studies have demonstrated that co-administration of 5-ASA can result in a higher 6-TGN level in patients with IBD on AZA or mercaptopurine,^16,44,45^ which was also confirmed in our study. Of the patients on AZA plus 5-ASA medications, there was an increase in the frequency of clinical leucopenia of 20% and in the 6-TGN level compared with baseline therapy with AZA alone.

It should be noted that there were several potential weaknesses in our study. It is better to use endoscopic and objective biomarkers to define response contrast to the high variability of CDAI. To avoid this weakness, the aim of this study only evaluate the relationship between response to steroid sparing and metabolite levels, the patients on AZA plus infliximab, on AZA for postoperative prophylaxis, and on AZA for endoscopic relapse after surgical resection were excluded from the analysis. Meanwhile, a response to steroid sparing was defined as resolution of symptoms upon complete withdrawal of all corticosteroids for at least 6 months.

## CONCLUSIONS

In summary, AZA metabolite measurements may provide clinicians with useful tools to optimize and individualize AZA therapy in IBD. The advantages of metabolite monitoring observed in this study include predicting leucopenia in the initial 3 months of AZA therapy and predicting a reasonable chance of successful clinical response in patients with CD. Another potential value of 6-TGN testing concerns the timing of the testing. Early testing (after 2–4 weeks of therapy) could identify patients likely to benefit from a dose adjustment. Although AZA should be dosed according to body weight, previous research has documented a poor correlation between dose on a per-kilogram basis and 6-TGN levels. Thus, even in patients prescribed the recommended dose of these medications, the measurement of 6-TGN levels may provide additional useful information. Patient with 6-TGN level >420 pmol/8 × 10^8^ RBC at week 2 could reduce AZA dose to avoid leucopenia, and increase drug dose for a successful clinical response if patient has 6-TGN level below 225 pmol/8 × 10^8^ RBC. A full blood count may be more important after 3 months of AZA therapy considering that 6-TGN is more appropriate to predict leucopenia in the initial 3 months of AZA therapy. This study also suggests that co-administration of 5-ASA with AZA results in a high frequency of leucopenia in patients, which could be associated with an increase in 6-TGN concentrations. Considering ethnic differences, a larger prospective study in different ethnic groups is now needed to verify this observation.

## References

[1] Dubinsky MC Yang H Hassard PV 6-MP metabolite profiles provide a biochemical explanation for 6-MP resistance in patients with inflammatory bowel disease. *Gastroenterology* 2002; 122:904–915. 11910342 10.1053/gast.2002.32420

[2] Fraser AG Orchard TR Jewell DP The efficacy of azathioprine for the treatment of inflammatory bowel disease: a 30 year review. *Gut* 2002; 50:485–489. 11889067 10.1136/gut.50.4.485 PMC1773162

[3] Sandborn W Sutherland L Pearson D Azathioprine or 6-mercaptopurine for inducing remission of Crohn's disease. *Cochrane Database Syst Rev* 2000; CD000545. 10796557 10.1002/14651858.CD000545

[4] Pearson DC May GR Fick GH Azathioprine and 6-mercaptopurine in Crohn disease. A meta-analysis. *Ann Intern Med* 1995; 123:132–142.R. 7778826 10.7326/0003-4819-123-2-199507150-00009

[5] Fangbin Z Xiang G Minhu C Should thiopurine methyltransferase genotypes and phenotypes be measured before thiopurine therapy in patients with inflammatory bowel disease? *Ther Drug Monit* 2012; 34:695–701. 23149442 10.1097/FTD.0b013e3182731925

[6] de Jong DJ Derijks LJ Naber AH Safety of thiopurines in the treatment of inflammatory bowel disease. *Scand J Gastroenterol Suppl* 2003; 239:69–72.Review. 14743886 10.1080/00855920310002726

[7] Krynetski EY Krynetskaia NF Yanishevski Y Methylation of mercaptopurine, thioguanine, and their nucleotide metabolites by heterologously expressed human thiopurine S-methyltransferase. *Mol Pharmacol* 1995; 47:1141–1147. 7603453

[8] Lennard L Van Loon JA Lilleyman JS Thiopurine pharmacogenetics in leukemia: correlation of erythrocyte thiopurine methyltransferase activity and 6-thioguanine nucleotide concentrations. *Clin Pharmacol Ther* 1987; 41:18–25. 3467886 10.1038/clpt.1987.4

[9] Lennard L Van Loon JA Weinshilboum RM Pharmacogenetics of acute azathioprine toxicity: relationship to thiopurine methyltransferase genetic polymorphism. *Clin Pharmacol Ther* 1989; 46:149–154. 2758725 10.1038/clpt.1989.119

[10] Anstey A Lennard L Mayou SC Pancytopenia related to azathioprine-an enzyme deficiency caused by a common genetic polymorphism: a review. *J R Soc Med* 1992; 85:752–756. 1494166 10.1177/014107689208501213 PMC1293765

[11] Ansari A Hassan C Duley J Thiopurine methyltransferase activity and the use of azathioprine in inflammatory bowel disease. *Aliment Pharmacol Ther* 2002; 16:1743–1750. 12269967 10.1046/j.1365-2036.2002.01353.x

[12] Gisbert JP Luna M Maté J Choice of azathioprine or 6-mercaptopurine dose based on thiopurine methyltransferase (TPMT) activity to avoid myelosuppression. A prospective study. *Hepatogastroenterology* 2006; 53:399–404. 16795981

[13] Colombel JF Ferrari N Debuysere H Genotypic analysis of thiopurine S-methyltransferase in patients with Crohn's disease and severe myelosuppression during azathioprine therapy. *Gastroenterology* 2000; 118:1025–1030. 10833476 10.1016/s0016-5085(00)70354-4

[14] Osterman MT Kundu R Lichtenstein GR Association of 6-thioguanine nucleotide levels and inflammatory bowel disease activity: a meta-analysis. *Gastroenterology* 2006; 130:1047–1053. 16618398 10.1053/j.gastro.2006.01.046

[15] Goldenberg BA Rawsthorne P Bernstein CN The utility of 6-thioguanine metabolite levels in managing patients with inflammatory bowel disease. *Am J Gastroenterol* 2004; 99:1744–1748. 15330913 10.1111/j.1572-0241.2004.30415.x

[16] Lowry PW Franklin CL Weaver AL Measurement of thiopurine methyltransferase activity and azathioprine metabolites in patients with inflammatory bowel disease. *Gut* 2001; 49:665–670. 11600469 10.1136/gut.49.5.665 PMC1728511

[17] Jharap B Seinen ML de Boer NK Thiopurine therapy in inflammatory bowel disease patients: Analyses of two 8-year intercept cohorts. *Inflamm Bowel Dis* 2010; 16:1541–1549. 20155846 10.1002/ibd.21221

[18] Hindorf U Lindqvist M Peterson C Pharmacogenetics during standardised initiation of thiopurine treatment in inflammatory bowel disease. *Gut* 2006; 55:1423–1431. 16543290 10.1136/gut.2005.074930 PMC1856436

[19] Dubinsky MC Lamothe S Yang HY Pharmacogenomics and metabolite measurement for 6-mercaptopurine therapy in inflammatory bowel disease. *Gastroenterology* 2000; 118:705–713. 10734022 10.1016/s0016-5085(00)70140-5

[20] Roblin X Peyrin-Biroulet L Phelip JM A 6-thioguanine nucleotide threshold level of 400 pmol/8 x 10 (8) erythrocytes predicts azathioprine refractoriness in patients with inflammatory bowel disease and normal TPMT activity. *Am J Gastroenterol* 2008; 103:3115–3122. 19086961 10.1111/j.1572-0241.2008.01743.x

[21] Lennard-Jones JE Classification of inflammatory bowel disease. *Scand J Gastroenterol Suppl* 1989; 170:2–6.discussion 16-19. 2617184 10.3109/00365528909091339

[22] Best WR Becktel JM Singleton JW Development of a Crohn's disease activity index. National Cooperative Crohn's Disease Study. *Gastroenterology* 1976; 70:439–444. 1248701

[23] Sutherland LR Martin F Greer S 5-Aminosalicylic acid enema in the treatment of distal ulcerative colitis, proctosigmoiditis, and proctitis. *Gastroenterology* 1987; 92:1894–1898. 3569765 10.1016/0016-5085(87)90621-4

[24] Ding L Zhang FB Liu H Hypoxanthine guanine phosphoribosyltransferase activity is related to 6-thioguanine nucleotide concentrations and thiopurine-induced leukopenia in the treatment of inflammatory bowel disease. *Inflamm Bowel Dis* 2012; 18:63–73. 21381155 10.1002/ibd.21676

[25] Dervieux T Boulieu R Simultaneous determination of 6-thioguanine and methyl 6-mercaptopurine nucleotides of azathioprine in red blood cells by HPLC. *Clin Chem* 1998; 44:551–555. 9510860

[26] Lennard L Singleton HJ High-performance liquid chromatographic assay of the methyl and nucleotide metabolites of 6-mercaptopurine: quantitation of red blood cell 6-thioguanine nucleotide, 6-thioinosinic acid and 6-methylmercaptopurine metabolites in a single sample. *J Chromatogr* 1992; 583:83–90. 1484095 10.1016/0378-4347(92)80347-s

[27] Xin HW Xiong H Wu XC Relationships between thiopurine S-methyltransferase polymorphism and azathioprine-related adverse drug reactions in Chinese renal transplant recipients. *Eur J Clin Pharmacol* 2009; 65:249–255. 19048245 10.1007/s00228-008-0589-0

[28] Cao Q Zhu Q Shang Y Thiopurine methyltransferase gene polymorphisms in Chinese patients with inflammatory bowel disease. *Digestion* 2009; 79:58–63. 19252404 10.1159/000205268

[29] Hindorf U Lindqvist M Hildebrand H Adverse events leading to modification of therapy in a large cohort of patients with inflammatory bowel disease. *Aliment Pharmacol Ther* 2006; 24:331–342. 16842460 10.1111/j.1365-2036.2006.02977.x

[30] Schütz E Gummert J Mohr FW Should 6-thioguanine nucleotides be monitored in heart transplant recipients given azathioprine? *Ther Drug Monit* 1996; 18:228–233. 8738760 10.1097/00007691-199606000-00002

[31] Gupta P Gokhale R Kirschner BS 6-mercaptopurine metabolite levels in children with inflammatory bowel disease. *J Pediatr Gastroenterol Nutr* 2001; 33:450–454. 11698762 10.1097/00005176-200110000-00006

[32] McGovern DP Travis SP Duley J Azathioprine intolerance in patients with IBD may be imidazole-related and is independent of TPMT activity. *Gastroenterology* 2002; 122:838–849. 11878296 10.1053/gast.2002.32124

[33] Cuffari C Hunt S Bayless T Utilisation of erythrocyte 6-thioguanine metabolite levels to optimise azathioprine therapy in patients with inflammatory bowel disease. *Gut* 2001; 48:642–646. 11302961 10.1136/gut.48.5.642 PMC1728278

[34] Jewell DP Truelove SC Azathioprine in ulcerative colitis: final report on controlled therapeutic trial. *Br Med J* 1974; 4:627–630. 4441827 10.1136/bmj.4.5945.627 PMC1612983

[35] Rosenberg JL Wall AJ Levin B A controlled trial of azathioprine in the management of chronic ulcerative colitis. *Gastroenterology* 1975; 69:96–99. 1097295

[36] Kirk AP Lennard-Jones JE Controlled trial of azathioprine in chronic ulcerative colitis. *Br Med J (Clin Res Ed)* 1982; 284:1291–1292. 10.1136/bmj.284.6325.1291 PMC1498148 6803944

[37] Zelinkova Z Derijks LJ Stokkers PC Inosine triphosphate pyrophosphatase and thiopurine s-methyltransferase genotypes relationship to azathioprine-induced myelosuppression. *Clin Gastroenterol Hepatol* 2006; 4:44–49. 16431304 10.1016/j.cgh.2005.10.019

[38] Ansari A Arenas M Greenfield SM Prospective evaluation of the pharmacogenetics of azathioprine in the treatment of inflammatory bowel disease. *Aliment Pharmacol Ther* 2008; 28:973–983. 18616518 10.1111/j.1365-2036.2008.03788.x

[39] Weinshilboum RM Sladek SL Mercaptopurine pharmacogenetics: monogenic inheritance of erythrocyte thiopurine methyltransferase activity. *Am J Hum Genet* 1980; 32:651–662. 7191632 PMC1686086

[40] Schaeffeler E Fischer C Brockmeier D Comprehensive analysis of thiopurine S-methyltransferase phenotype-genotype correlation in a large population of German-Caucasians and identification of novel TPMT variants. *Pharmacogenetics* 2004; 14:407–417. 15226673 10.1097/01.fpc.0000114745.08559.db

[41] Lennard L TPMT in the treatment of Crohn's disease with azathioprine. *Gut* 2002; 51:143–146. 12117866 10.1136/gut.51.2.143 PMC1773327

[42] Krynetski EY Evans WE Genetic polymorphism of thiopurine S-methyltransferase: molecular mechanisms and clinical importance. *Pharmacology* 2000; 61:136–146. 10971199 10.1159/000028394

[43] Zhang JP Guan YY Wu JH Phenotyping and genotyping study of thiopurine S-methyltransferase in healthy Chinese children: a comparison of Han and Yao ethnic groups. *Br J Clin Pharmacol* 2004; 58:163–168. 43. 15255798 10.1111/j.1365-2125.2004.02113.x PMC1884582

[44] de Boer NK Wong DR Jharap B Dose-dependent influence of 5-aminosalicylates on thiopurine metabolism. *Am J Gastroenterol* 2007; 102:2747–2753. 17764493 10.1111/j.1572-0241.2007.01511.x

[45] Hande S Wilson-Rich N Bousvaros A 5-aminosalicylate therapy is associated with higher 6-thioguanine levels in adults and children with inflammatory bowel disease in remission on 6-mercaptopurine or azathioprine. *Inflamm Bowel Dis* 2006; 12:251–257. 16633046 10.1097/01.MIB.0000206544.05661.9f

